# Metagenomes, Metatranscriptomes, and Metagenome-Assembled Genomes from Chesapeake and Delaware Bay (USA) Water Samples

**DOI:** 10.1128/MRA.00262-21

**Published:** 2021-05-27

**Authors:** Mir Alvee Ahmed, Shen Jean Lim, Barbara J. Campbell

**Affiliations:** a Department of Biological Sciences, Clemson University, Clemson, South Carolina, USA; Indiana University, Bloomington

## Abstract

Here, we present 36 metagenomes, 59 metatranscriptomes, and 373 metagenome-assembled genomes (MAGs) from Chesapeake and Delaware Bay water samples. This data set will be useful for studying microbial biogeochemical cycling in estuaries.

## ANNOUNCEMENT

Estuaries are productive aquatic environments harboring diverse flora, fauna, and microbial communities important for global carbon and nutrient cycling (1, 2). Omics analyses of estuary-associated bacteria will reveal associations between bacterial communities, functional composition, and environmental variation. Three cruises and two cruises along longitudinal transects of the Delaware Bay (DEBay) and Chesapeake Bay (CPBay), two geographically close estuaries with contrasting environmental gradients, were completed in 2014 and 2015, respectively, aboard R/V *Sharp.* Surface (∼1.5 m below the seafloor [mbsf]) water samples were collected using a rosette sampler with associated conductivity-temperature-depth (CTD) profiles. The sampling scheme (3), environmental measurements, and bacterial production measurements (4–7) were described previously (4–7) and archived (https://www.bco-dmo.org/dataset/565451).

Cells were separated as large- and small-cell-size fractions by passing water samples through 0.8- and 0.22-μm-pore-size filters. Nucleic acids were extracted from size-fractionated cells using the Allprep DNA/RNA minikit (Qiagen, Valencia, CA, USA) (3). A total of 36 (12 from CPBay and 24 from DEBay) metagenomic and 59 (24 from CPBay and 35 from DEBay) metatranscriptomic libraries were prepared using the TruSeq library preparation kit (Illumina) and sequenced by the Joint Genome Institute on the Illumina HiSeq 2000 platform at 2 × 150 bp, as described previously (3). Two DEBay metagenomes (DEBay_Spr_30_<0.8_DNA and DEBay_Sum_22_D_<0.8_DNA) were also sequenced in-house with the Nanopore rapid sequencing kit (Oxford Nanopore Technologies, Kidlington, Oxfordshire, UK) on a MinION flow cell (R9.4 nanopores) with a MinION Mk1B sequencer. MinKNOW was used for basecalling (8). Sequencing statistics are in Table 1 (metagenomes) and online at https://doi.org/10.6084/m9.figshare.14173664 (metatranscriptomes).

**TABLE 1:** Accession numbers and characteristics of metagenomes from the Chesapeake and Delaware Bay water samples

Metagenome	NCBI BioSample no.	Collection date (yr-mo-day), time	No. of raw reads	No. of paired reads	No. of contigs	Total length (bp)	*N*_50_ (bp)
CPBay_Spr_0.1_0.8_DNA	SAMN06266052	2015-4-11, 8:54	281,517,088	270,649,460	38,004	64,955,483	1,606
CPBay_Spr_15_0.2_DNA	SAMN06264353	2015-4-13, 7:00	120,796,538	106,995,332	67,010	175,958,607	3,173
CPBay_Spr_15_0.8_DNA	SAMN06266053	2015-4-13, 7:00	48,340,218	45,866,608	102,184	307,564,961	4,092
CPBay_Spr_31_0.2_DNA	SAMN06264351	2015-4-16, 7:25	74,037,960	72,685,680	95,167	260,760,381	3,425
CPBay_Spr_31_0.8_DNA	SAMN06264995	2015-4-16, 7:25	173,012,398	158,595,630	147,394	376,160,656	3,055
CPBay_Sum_0.6_0.2_DNA	SAMN06266060	2015-8-17, 10:50	141,437,090	123,369,312	244,783	609,304,882	2,860
CPBay_Sum_0.6_0.8_DNA	SAMN06265975	2015-8-17, 10:50	306,249,226	288,883,072	165,685	439,027,102	3,214
CPBay_Sum_15_0.2_DNA	SAMN06265905	2015-8-20, 10:54	52,780,740	50,636,990	86,423	198,558,572	2,459
CPBay_Sum_15_0.8_DNA	SAMN06266062	2015-8-20, 10:54	37,888,140	36,283,452	94,006	245,336,553	3,092
CPBay_Sum_20_0.8_DNA	SAMN06264994	2015-8-21, 10:58	49,252,868	46,975,502	96,381	226,955,816	2,597
CPBay_Sum_27_0.2_DNA	SAMN06265909	2015-8-22, 12:05	51,275,568	49,528,260	89,461	204,236,447	2,450
CPBay_Sum_27_0.8_DNA	SAMN06265908	2015-8-22, 12:05	44,125,638	42,165,180	104,303	252,367,039	2,714
DEBay_Fall_0.3_<0.8_DNA	SAMN06267360	2014-11-1, 10:58	52,987,214	49,649,484	80,360	186,561,286	2,537
DEBay_Fall_0.3_>0.8_DNA	SAMN06343911	2014-11-1, 10:58	53,877,142	45,891,604	15,941	27,685,441	1,684
DEBay_Fall_15_<0.8_DNA	SAMN06343912	2014-11-2, 10:53	60,854,638	57,122,564	77,305	181,237,773	2,621
DEBay_Fall_15_>0.8_DNA	SAMN06343913	2014-11-2, 10:53	51,898,766	48,359,082	100,334	255,186,815	2,991
DEBay_Fall_30_<0.8_DNA	SAMN06343914	2014-11-2, 11:00	43,142,140	41,842,196	99,481	216,180,152	2,284
DEBay_Fall_30_>0.8_DNA	SAMN06267361	2014-11-3, 11:00	55,485,148	52,093,204	84,654	198,454,970	2,562
DEBay_Spr_0.19_<0.8_DNA	SAMN06343915	2014-3-19, 7:15	202,927,872	185,316,006	12,660	22,762,283	1,744
DEBay_Spr_0.19_>0.8_DNA	SAMN06343916	2014-3-19, 7:15	49,126,458	46,738,750	49,212	88,800,034	1,752
DEBay_Spr_20_<0.8_DNA	SAMN06343917	2014-3-21, 10:00	46,744,568	45,198,034	189,456	516,684,036	3,292
DEBay_Spr_20_>0.8_DNA	SAMN06343918	2014-3-21, 10:00	226,242,708	206,597,410	67,590	179,499,373	3,218
DEBay_Spr_30_<0.8_DNA	SAMN06267362	2014-3-22, 10:00	66,038,034	62,436,828	69,437	203,303,769	4,075
DEBay_Spr_30_<0.8_DNA^*a*^	SAMN06267362	2014-3-22, 10:00	29,390	NA^*b*^	25,608	100,659,831	6,542
DEBay_Spr_30_>0.8_DNA	SAMN06267363	2014-3-22, 10:00	61,482,122	57,197,948	78,393	222,454,675	3,684
DEBay_Sum_0.19_D_<0.8_DNA	SAMN06343919	2014-8-28, 11:04	47,505,130	45,015,264	80,761	193,840,000	2,670
DEBay_Sum_0.19_D_>0.8_DNA	SAMN06343920	2014-8-28, 11:04	124,114,698	118,522,690	226,532	556,422,472	2,786
DEBay_Sum_0.19_N_<0.8_DNA	SAMN06343921	2014-8-27, 22:57	108,517,694	103,811,934	180,641	368,341,091	2,079
DEBay_Sum_0.19_N_>0.8_DNA	SAMN06343922	2014-8-27, 22:57	78,727,834	71,831,928	70,693	152,225,938	2,242
DEBay_Sum_22_D_<0.8_DNA	SAMN06343923	2014-8-31, 11:02	49,987,622	48,008,464	164,532	359,332,827	2,316
DEBay_Sum_22_D_<0.8_DNA^*a*^	SAMN06343923	2014-8-31, 11:02	320,000	NA^*b*^	296,849	919,368,458	5,297
DEBay_Sum_22_D_>0.8_DNA	SAMN06343924	2014-8-31, 11:02	85,810,096	77,867,130	83,951	239,126,622	3,706
DEBay_Sum_22_N_<0.8_DNA	SAMN06343925	2014-8-30, 23:01	73,865,228	67,739,506	140,456	349,551,209	2,852
DEBay_Sum_22_N_>0.8_DNA	SAMN06343926	2014-8-30, 23:01	80,573,046	74,251,374	108,312	297,387,441	3,429
DEBay_Sum_29_D_<0.8_DNA	SAMN06343927	2014-9-1, 11:00	42,251,814	40,672,184	141,375	341,203,775	2,715
DEBay_Sum_29_D_>0.8_DNA	SAMN06343928	2014-9-1, 11:00	75,955,068	70,195,454	87,345	220,428,935	2,917
DEBay_Sum_29_N_<0.8_DNA	SAMN06267364	2014-8-31, 23:01	105,157,540	99,789,694	10,675	25,193,692	2,607
DEBay_Sum_29_N_>0.8_DNA	SAMN06343929	2014-8-31, 23:01	70,891,734	64,601,422	116,166	287,901,815	2,870

a Nanopore sequences.

b NA, not available.

Prior to assembly, Cutadapt v1.11 and Sickle v1.33 were used to remove adapters from and quality trim (Q = 30) Illumina-sequenced reads (9). Nanopore-sequenced reads were not error corrected or trimmed prior to hybrid assembly, as recommended by hybridSPAdes v3.11.1, because they were used only for gap closure and repeat resolution (10). Read qualities pre- and posttrimming were assessed with FastQC v0.11.5 (Babraham Bioinformatics, 2010). Metagenomic assemblies were performed using the default parameters of metaSPAdes v3.11.1 (11) with increased memory allocation (--meta --m 450) and evaluated using MetaQUAST, v5.0.2 (12) (Table 1).

For binning, trimmed reads from each Illumina-sequenced library were mapped to contigs ≥2,000 bp in the corresponding metagenome using the default parameters (end-to-end mode) of Bowtie 2 v2.2.7 (13). Alignments converted to binary alignment map (BAM) format with SAMtools v0.1.19 (13, 14) were binned into 373 metagenome-assembled genomes (MAGs) using the default parameters of MetaBAT2 v2.10.2 (15). MAG statistics, including GC content, size, completeness, and contamination, were assessed by CheckM v1.0.16 (16) and Anvi’o v6.2 and v7 (17–19). Coassembled sequences of both size fractions from the same water sample were binned when separate binning did not give useful MAGs. A subset of 364 MAGs (https://doi.org/10.6084/m9.figshare.14179448) with >80% completion and <5% contamination were taxonomically annotated using Anvi’o and Microbial Genome Atlas (MiGA) v0.7.26.2 (20). They belonged to bacterial orders *Actinomycetales* (*n* = 7), *Burkholderiales* (*n* = 31), *Flavobacteriales* (*n* = 55), *Microtrichales* (*n* = 39), *Nanopelagicales* (*n* = 11), *Pelagibacterales* (*n* = 31), *Pseudomonadales* (*n* = 26), *Rhodobacterales* (*n* = 28), and *Synechococcales* (*n* = 13), as well as the archaeal phyla *Crenarchaeota* (*n* = 5) and *Euryarchaeota* (*n* = 2).

### Data availability.

The metagenomes, metatranscriptomes, and MAGs are available on NCBI under the umbrella project PRJNA432171.
